# Monte Carlo modelling of a compact CZT-based gamma camera with application to ^177^Lu imaging

**DOI:** 10.1186/s40658-022-00463-1

**Published:** 2022-05-08

**Authors:** Daniel Roth, Erik Larsson, Michael Ljungberg, Katarina Sjögreen Gleisner

**Affiliations:** 1 Medical Radiation Physics, Lund, Lund University, Lund, Sweden; 2 Department of Radiation Physics, Skåne University Hospital, Lund, Sweden

**Keywords:** CZT, Handheld gamma camera, Molecular imaging, Monte Carlo

## Abstract

**Background:**

Semiconductor gamma-camera systems based on cadmium zinc telluride (CZT) detectors present new challenges due to an energy-response that includes effects of low-energy tailing. In particular, such energy tails produce effects that need to be considered when imaging radionuclides with multiple emissions such as $^{177}{\mathrm {Lu}}$. Monte Carlo simulation can be used to investigate the behaviour of such systems and optimise their use, provided that the detector model closely reflects the real physical detector. The aim of this work is to develop a CZT model applicable for simulation of CZT-based gamma cameras.

**Methods:**

The equations describing the charge transport and signal induction are considered in three dimensions and are solved numerically, and the CZT model is then realised by coupling the detector-response to the photon-transport handled by the SIMIND Monte Carlo program. The CZT model is tuned to reproduce experimentally measured energy spectra of a hand-held gamma camera system for multiple radionuclides ($^{99\mathrm {m}}{\mathrm {Tc}}$, $^{123}{\mathrm {I}}$ and $^{177}{\mathrm {Lu}}$) and parallel-hole collimators (MEGP, LEHR) as well as an uncollimated system.

**Results:**

Overall, the model results agree well with measurements across the range of experimental conditions. The applicability of the model is demonstrated by separating energy spectra into components to investigate the interference of high-energy photons on lower energy-windows, where pronounced effects of low-energy tailing for $^{177}{\mathrm {Lu}}$ are observed.

**Conclusions:**

The developed model provides understanding of the specifics of the camera response and is expected to be helpful for future optimisation of gamma camera applications.

**Supplementary Information:**

The online version contains supplementary material available at 10.1186/s40658-022-00463-1.

## Background

Semiconductor-based gamma cameras utilising cadmium zinc telluride (CZT) are becoming increasingly common in clinical applications [1–7]. Such cameras generally have favourable properties compared to conventional Anger cameras, such as better energy and intrinsic spatial resolution. The detector modules incorporated in CZT cameras are more compact than the typical photomultiplier tube arrays used in conventional Anger-cameras, and provide useful fields of view (FOVs) equal to the full detector-area. These aspects offer additional advantages and opportunities, as they allow for novel camera designs with efficient measurement geometries (see e.g.  [1, 3, 6]). The two major vendors for clinical CZT cameras are GE Healthcare and Spectrum Dynamics Medical, that offer SPECT systems like the Discovery NM530c [1], D-SPECT [2], Discovery 870 CZT [5], Veriton [6] and StarGuide [7]. CZT-based cameras do, however, typically present undesirable spectrum features known as low-energy tails, which result from poor charge carrier transport properties (low mobilities or short lifetimes), particularly for holes, an issue prevalent among compound semiconductors [8]. Special electrode configurations such as pixelated anodes [9] reduce the tails, but do not eliminate them completely. The low-energy tails present problems for imaging of certain radionuclides where high-energy photon emissions contribute to the energy windows set over emissions of lower energy [10, 11].

$^{177}{\mathrm {Lu}}$ is a $\beta ^-$-emitter used within radionuclide therapy. Its applications include [$^{177}{\mathrm {Lu}}$]Lu-DOTA-TATE for neuroendocrine tumours [12] and [$^{177}{\mathrm {Lu}}$]Lu-PSMA for prostate cancer [13, 14]. $^{177}{\mathrm {Lu}}$ imaging is possible through several photon emissions with suitable energies and yields. The most prominent emissions are 54.6 keV, 55.8 keV, 112.9 keV and 208.4 keV, and the near-indistinguishable combination of the first two emissions is henceforth referred to as 55 keV. For CZT-detectors, the low-energy tailing means that the high-energy photon emissions will contribute to lower energy windows (mainly 113 keV and 55 keV windows). Imaging of $^{177}{\mathrm {Lu}}$ therefore requires additional considerations compared to radionuclides with simpler, near-monoenergetic, spectra. Implications of low-energy tailing have been studied previously, mainly regarding dual-isotope acquisitions of $^{99\mathrm {m}}{\mathrm {Tc}}$+$^{201}{\mathrm {Tl}}$ and $^{99\mathrm {m}}{\mathrm {Tc}}$+$^{123}{\mathrm {I}}$ [10, 15–18].

Monte Carlo simulation is a tool used to investigate the performance and improve the understanding of detector systems. A major advantage is that effects that are difficult to investigate by experimental measurements can be readily isolated and studied. Complete modelling of a detector measurement requires that a number of processes are addressed, including the radiation emission and transport, the interactions in the detector, the generation of electrical output-signals from the detector and the post-processing of these signals. These processes can be modelled at different levels of detail, with a degree of complexity that depends on the targeted detector system and its application [19]. Monte Carlo modelling of scintillation-based gamma cameras is well-established, whereas the modelling of their semiconductor-based counterparts is a newer field in which more detailed considerations of the signal-generation process are required. For Anger-type scintillation-camera models it may suffice with a simplified scintillation light collection model with a fixed position-independent collection fraction, whereas for semiconductor-based detectors the response to the motions of the created electron-hole pairs is position-dependent and needs to be considered. This position dependence can be modelled with different degree of complexity, ranging from simpler one-dimensional approximations [20] to comprehensive three-dimensional models [21], or somewhere in-between the two [22, 23].

The number of Monte Carlo programs that readily includes modelling of CZT imaging is currently limited, and those that exist sometimes rely on simplified analytical expressions whose validity may affect the accuracy. In addition, the use of CZT signal generation models requires knowledge of parameters and material properties that may be impractical to measure directly by end users of CZT cameras. Consequently, reaching the point where simulations agree sufficiently well with measurements may be challenging. In particular, our initial attempts with a simplified CZT model, presented in [23], for a small hand-held gamma camera [3, 11] gave poor agreements when evaluated across multiple parallel-hole collimators and radionuclides. Especially, accurate simulation of $^{177}{\mathrm {Lu}}$ with its three energy peaks has proved to be challenging, possibly due to the validity of the assumptions and approximations made by the employed model.

Thus, the aim of this work was to develop a CZT model for imaging and spectroscopy, combine it with the SIMIND Monte Carlo program [24] for photon transport, and investigate a practical method of tuning it against experimental measurements with the hand-held camera. This is a system centred around a single CZT-module [25], whose specifications are identical or similar to modules found in full-size gamma cameras (e.g.  [1, 2, 4, 5]), and the developed model should therefore be relevant for such systems as well (personal communication, Aharon Peretz, General Electric, retired).

## Materials and methods

### Charge transport and signal induction

For CZT-based semiconductor detectors, the strong interaction-position-dependence of the detector signal can be calculated with the Shockley–Ramo theorem [26–28], as outlined in detail in Additional file 1: Appendix A. The detector response can be calculated from the equations

1 $$\begin{aligned}&\nabla ^2 \varphi = \frac{\rho }{\varepsilon }, \end{aligned}$$

2 $$\begin{aligned}&\nabla ^2 \varphi _{k} = 0,\end{aligned}$$

3 $$\begin{aligned}&\frac{\partial x}{\partial t} = \pm \nabla \cdot (\mu _x x\nabla \varphi ) + \nabla \cdot (D_x \nabla x) + G_x - R_x, \end{aligned}$$

4 $$\Delta Q_{x,k}(t) = q \cdot \int _{0}^{t} \int _{\mathbf {r}\in \Omega } x\left( \mathbf {r},t'\right) \cdot \mu _x \cdot \nabla \varphi \left( \mathbf {r}\right) \cdot \nabla \varphi _{k}\left( \mathbf {r}\right) {\rm d}\Omega {\rm d}t'.$$

Equation 1 is Gauss’s law, which is solved for the electric potential $\varphi$ in the detector crystal, $\rho$ is the charge density and $\varepsilon$ is the permittivity of the detector crystal [29, 30].

Equation 2 gives the so-called weighting potential $\varphi _{k}$ for electrode *k* that is attached to the detector crystal.

Equation 3 describes the motion of a cloud of excess charge carriers [19, 30, 31], which for semiconductors can represent electrons ($x=n$) or holes ($x=p$). The sign of the first term is negative for electrons and positive for holes, $\mu _x$ is the charge carrier mobility, $D_x$ is the diffusion constant [32], and $G_x$ and $R_x$ express the creation of new charges and recombination of existing charges, respectively. Charge recombination can be described as $R_x = x/\tau _x$, where $\tau _x$ is the charge carrier lifetime. A typical generation term in the context of radiation interactions is the creation of a single point-like charge generated at an arbitrary point $\mathbf {r}_0$ at time $t=0$; $G_x = \delta \left( \left| \mathbf {r}-\mathbf {r}_0\right| \right) \cdot \delta \left( t\right)$, where $\delta (\cdot )$ is the Dirac delta function.

Equation 4 is the Shockley–Ramo theorem, and gives the charge $\Delta Q$ that is induced on electrode *k* due to the motion of a charge cloud $x\left( \mathbf {r},t\right)$, where $\Omega$ is the crystal volume [19, 22, 30, 31].

The induced charge can be considered as a function of the integration time *t*, and the point $\mathbf {r}_0$ where the charge was initially created, i.e.  $\Delta Q_{x,k}(t) = \Delta Q_{x,k}(\mathbf {r}_0,t)$. The charge induction efficiency (CIE) $\eta$ is a quantity that summarises the detector response as function of interaction position, and is defined as $\eta _{x,k}\left( \mathbf {r}_0,t\right) = Q_{x,k}(\mathbf {r}_0,t) / |q|$.

Calculation of $x(\mathbf {r},t)$ and $\eta _{x,k}\left( \mathbf {r}_0,t\right)$ for a large number of starting positions $\mathbf {r}_0$ directly using Eqs. 3 and 4 is computationally expensive. A more efficient method for computing $\eta$ is to use an adjoint method [19, 31], which introduces the equation

5 $$\begin{aligned} \frac{\partial x^+}{\partial t} = \mp \mu _x\nabla \varphi \cdot \nabla x^+ + \nabla \cdot (D_x \nabla x^+) + G_x^+ - x^+/\tau _x, \end{aligned}$$

where the sign of the first term is positive for electrons and negative for holes. With $G_x^+$ defined as $G_x^+ = \mu _x \nabla \varphi \cdot \nabla \varphi _{k}$ it can be shown that $x^+(\mathbf {r},t) = \eta _{x,k}\left( \mathbf {r},t\right)$ [19, 31], meaning that $\eta _{x,k}\left( \mathbf {r},t\right)$ can be determined for all starting positions by solving Eq. 5 once.

### The hand-held camera

The camera used was a CrystalCam hand-held gamma camera (Crystal Photonics GmbH, Berlin, Germany). The primary application for the instrument is sentinel lymph node localisation with $^{99\mathrm {m}}{\mathrm {Tc}}$, but other applications have been evaluated as well [3, 11]. The camera uses a single OMS40G256 CZT module (Orbotech Medical Solutions Ltd., Israel, now GE Healthcare) for imaging, with a crystal of dimensions $39 \times 39 \times 5$ mm$^{3}$. One side of the crystal is covered by a continuous cathode, and the opposing side by a $16 \times 16$ array of anode elements with a 2.46 mm pitch and a contact pad size of $1.86 \times 1.86$ mm$^{2}$. A 600 V potential is applied over the crystal, and the electrode properties are specified as ohmic. Further descriptions of similar modules are given by Vadawale et al. [33] and Kotoch et al. [25].

Collimators used were low energy high resolution (LEHR) and medium energy general purpose (MEGP), and also an open-field cover (OPEN) that has an air-cavity in place of collimating material. The collimators have holes matched one-to-one with the anodes of the detector. Collimator specifications are given in Table 1.

**Table 1:** Collimator dimensions

Name	Hole length	Wall thickness	Hole width	Material	Hole shape
LEHR	22.6	0.23	2.23	Lead	Square
MEGP	11.5	0.96	1.50	Lead	Circular

All values are given in mm. Table adapted from Roth et al. [11]

The camera can be used in low-energy mode, covering an energy interval of approximately 40 to 250 keV and high-energy mode, covering 40 to 1250 keV. Only the low-energy mode was used, as the high-energy mode exhibits greater inhomogeneities and is less suitable for imaging. The acquired data were stored in a table of spectra, stating for each anode element the number of counts as function of energy according to the manufacturers energy calibration, including equally spaced energy bins between 0 keV and 250 keV with a bin width of 0.1 keV.

### Experimental measurements

**Fig. 1:**
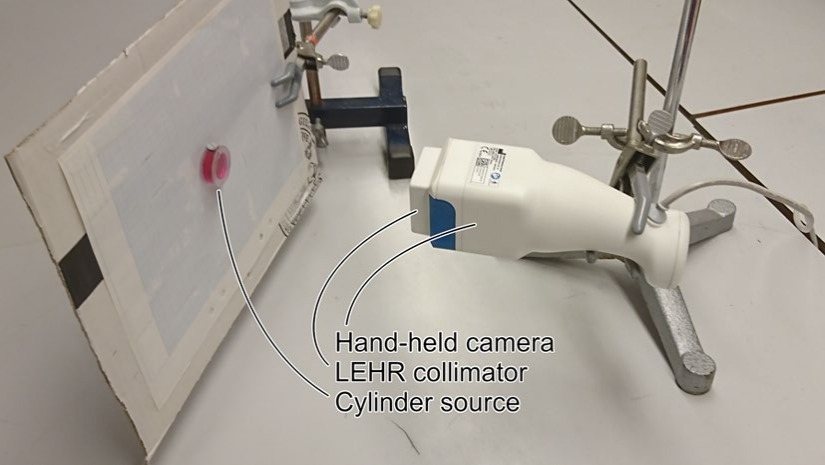
Illustration of the measurement setup with the hand-held camera and the cylindrical phantom. The phantom was filled with a magenta dye to visualise the inner cavity

Reference measurements were made to tune the detector model. A resealable phantom was used, consisting of a cylindrical cavity (20 mm diameter, 8 mm height) with PMMA walls (1 mm top and bottom thickness, 5 mm radial thickness) [11]. Spectra were acquired with the following radionuclides and collimators: $^{177}{\mathrm {Lu}}$ (MEGP, LEHR), $^{99\mathrm {m}}{\mathrm {Tc}}$ (MEGP, LEHR, OPEN) and $^{123}{\mathrm {I}}$ (MEGP, LEHR, OPEN). A traceable Secondary Standard Dose Calibrator (Southern Scientific, Henfield, United Kingdom) was used to quantify the activity added to the phantom. Measurements were made with the phantom in air to minimise scatter and backscatter, with source-collimator distances of between 25 mm and 150 mm. Figure 1 illustrates the measurement setup.

The model was further evaluated by measurements of the system sensitivity as a function of distance for $^{177}{\mathrm {Lu}}$ with the MEGP and LEHR collimators. The setup for sensitivity measurements were similar to that of the reference measurements, using source-collimator distances from 0 mm up to 100 mm (MEGP) and 130 mm (LEHR). Energy windows were positioned over 55 keV (49.7 keV to 59.7 keV), 113 keV (100.5 keV to 120.8 keV) and 208 keV (193.8 keV to 216.7 keV), and the total count rates across the full FOV were recorded. For the measured sensitivity, a function was fitted following $c_0 + c_1 \cdot e^{-c_2 \cdot d}$, where *d* is the source-collimator distance [34].

### CIE map calculation

A procedure for calculating CIE maps was implemented in Python using arrays, sparse matrices and linear equation solvers from the NumPy [35] and SciPy [36] packages. The procedure considered a rectangular crystal volume in a Cartesian coordinate system oriented consistently with the convention used by SIMIND. The source-facing side of the crystal was fully covered by a cathode. The opposing side was covered by an array of rectangular anode elements, and the signal generation for one selected anode in this array was considered.

Finite difference approaches [37] were used to numerically estimate the solutions to differential equations 1, 2 and 5. For boundary-value problems (Eqs. 1 and 2), this yielded systems of linear equations which were solved using the loose generalised minimum residual algorithm [38].

The boundary conditions implemented for the differential equations were as follows: For the electric potential ($\varphi$), a specified potential difference (600 V) was applied between the anodes and the cathode. The weighting potential ($\varphi _k$) was, by definition, calculated with one selected electrode (electrode *k*) held at a potential of unity and all other electrodes at zero. For charge transport ($x \in \{n,p,n^+,p^+\}$), the condition $x=0$ was applied on ohmic surfaces and $\nabla x \cdot {\hat{n}} = 0$ was applied on insulating surfaces, where ${\hat{n}}$ is the surface normal [19].

For the inter-anode gaps, the camera’s technical description did not provide sufficient basis for a model implementation of the electrical and weighting potentials. In addition, it was not clear whether the signal contribution from holes was entirely negligible or not. Because of this lack of information, a number of alternative boundary conditions and configurations were implemented (Table 2) and tested as part of the model tuning (“Model tuning” section).

**Table 2:** Configurations and boundary conditions considered for the CIE calculation procedure

Component (equation number)		Alternative	Configuration	Effective number of tunable parameters$^\mathrm{b}$
Electric potential	(1)	A1	Uniform electric field	0
	(1)	A2$^\mathrm{a,c}$	$\nabla \varphi \cdot \hat{n} = 0$	0
	(1)	A3	Weighted average of the results from A1 and A2	1
Weighting potential	(2)	B1$^\mathrm{a}$	$\nabla \varphi _k \cdot \hat{n} = 0$	0
	(2)	B2	Inverse-distance weighted transition	2
Signal generation	(5)	C1	Electrons only	2
	(5)	C2	Electrons and holes	3

$^\mathrm{a}$Boundary condition applied on inter-anode gaps.

$^\mathrm{b}$Parameters are listed in Additional file 1: Appendix E.

$^\mathrm{c}$Alternative A2 was not used on its own and is only defined here to clarify the definition of A3

For the electric potential ($\varphi$), alternative A1 represented the commonly used assumption of a uniform electric field throughout the crystal [20, 22, 23, 39]. Alternative A2 instead assumed that the electric field lines start and end on electrodes, as expected for ideal dielectric materials [40, 41]. For A2, Eq. 1 was solved with charge density assumed negligible [42]. For imperfect materials with conducting surfaces, a fraction of the field lines may terminate in the inter-anode gaps [40–42], as emulated by alternative A3.

For the weighting potential ($\varphi _k$) a transition from unity to zero must occur in the gaps between the selected anode and its neighbours. A boundary condition involving the gradient and the surface normal is commonly applied [31, 39, 43, 44], represented by alternative B1. The inverse-distance weighting [45] method (alternative B2) has not been used previously, and was introduced to obtain way to parametrise and adjust the steepness of the lateral sides of the weighting potential and CIE. Additionally, a scale factor was introduced for alternative B2 to force the weighting potential to reach zero at the edge of the neighbouring anode or at some position within the gap, thus allowing for adjustment of the "width" of the anode’s sensitive area. Further details on alternative B2 are given in Additional file 1: Appendix B.

Signal generation from electrons only (C1) or both electrons and holes (C2) were considered. Equation 5 was implemented using a first-order upwinding scheme. The signal integration time was assumed to be long compared to the collection times and the lifetimes of the charge carriers, and the boundary-value problems arising from setting $\partial x^+/\partial t = 0$ were solved rather than integrating Eq. 5 forward in time.

Due to the periodic pattern of the anode elements, a number of calculated quantities were expected to be symmetric [40, 46, 47]. In particular, this was expected for the electric potential, the weighting potential and the CIE for anode elements near the centre of the detector. For each established plane of symmetry, only one side was considered when solving the equations, and all gradients across were set to zero.

To reduce the computational burden and get manageable calculation times, a compromise was necessary between the extent of the crystal region considered and the spatial discretisation step size. Thus, a $5 \times 5$ anode element neighbourhood centred around the selected anode was considered instead of the whole crystal, giving a step size of 20 ${\upmu \mathrm{m}}$.

Once all calculations were complete, the CIE map was written to a binary file for later use in the full detector model, including the CIE array, the coordinates associated with the array elements and a selection of parameter values ($\mu _x,\tau _x$ etc.) for book-keeping.

For consistency control, a CIE map was also calculated without charge diffusion in a parallel-plate geometry (single anode and cathode each covering the entire sides). For this geometry, the electric and weighting fields are uniform. With these conditions, the resulting CIE had a depth-dependence consistent with the Hecht-equation [48].

### Monte Carlo simulation of photon interactions

**Fig. 2:**
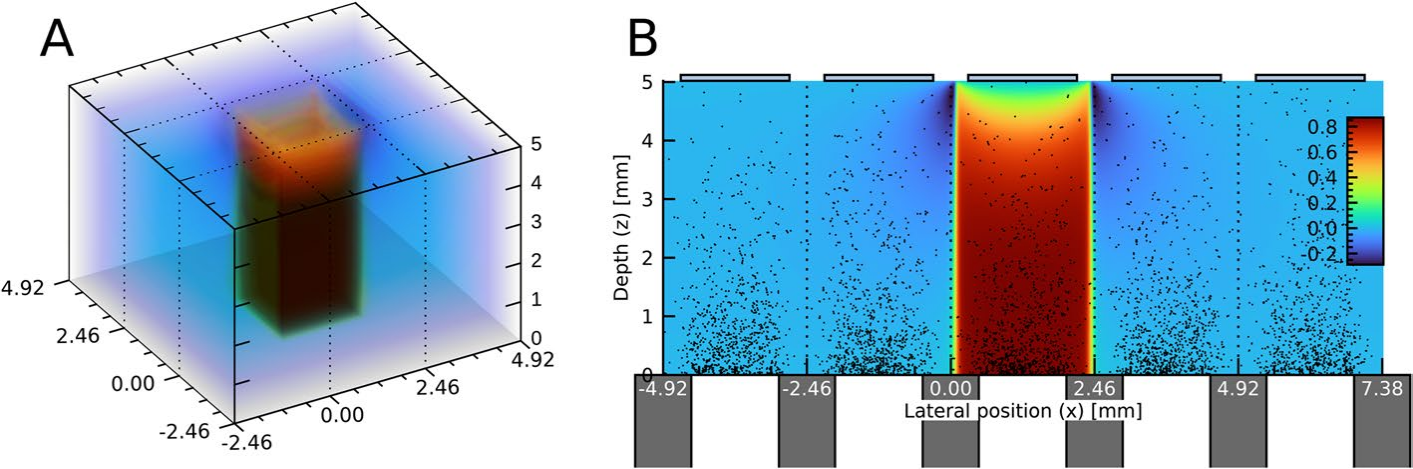
**A** Volume rendering of a CIE map ($\eta _k$). **B** Cross section of a CIE map with photon interaction positions from SIMIND overlaid as black points. The collimator septa (MEGP) are indicated at the bottom, and the decreased probability for interactions behind them can be noted. Dotted lines indicate the lateral centres of the anode gaps

The SIMIND Monte Carlo program [24] was used to generate sets of realistic photon interactions in the detector crystal. This was made for each of the physical measurements (“Experimental measurements” section). The collimators were simulated with full consideration of the collimator hole geometry and holes were aligned one-to-one with the detector anodes, to properly model the decreased likelihood of interactions above the gaps between the anodes. This collimator effect is visible in Fig. 2. Each photon interaction in the detector crystal was logged in a listmode format, including information about the interaction type (photoelectric absorption, Compton scatter, elastic scatter), interaction coordinates, deposited energy and variance-reduced photon weight [49]. The events originating from different photon histories were recorded as separate entries in the listmode file.

### Full detector model

A detector model mimicking the hand-held camera system was implemented in IDL (Interactive Data Language, Harris Geospatial Solutions Inc.). The basic building blocks were individual anode elements, which processed events separately from one-another. A CIE map ($\eta _k$) was associated to each element, and was defined according to

6 $$\begin{aligned} \eta _k(\mathbf {r}) = \eta _\mathrm {joint}(\mathbf {r} + \mathbf {r}_k-\mathbf {r}_\mathrm {joint}), \end{aligned}$$

where $\eta _\mathrm {joint}$ was generated using the procedure in “CIE map calculation” section. This joint CIE map, implemented in the form of a discrete three-dimensional array, was calculated for a central anode element ($\mathbf {r}_k = \mathbf {r}_\mathrm {joint}$). The associated maps for all other anode elements ($\mathbf {r}_k \ne \mathbf {r}_\mathrm {joint}$) were determined by applying suitable translations (Eq. 6). The full detector model was obtained by creating a $16 \times 16$ array of individual anode elements.

The model used the listmode files generated by SIMIND as input. For each photon history *H*, the total response $E_{\mathrm {out},k}$ for each anode element *k* was calculated according to

7 $$\begin{aligned} E_{\mathrm {out},k} = \sum _{i \in H} \eta _k\left( \mathbf {r}_i\right) \cdot E_i, \end{aligned}$$

where *i* denotes an individual photon interaction event for which the energy $E_i$ is deposited at a point $\mathbf {r}_i$. Tri-linear interpolation within the CIE array was used to evaluate $\eta _k\left( \mathbf {r}_i\right)$. This coupling between a calculated CIE map and interactions from SIMIND is illustrated in Fig. 2.

Following Eq. 7, each photon history yielded 256 separate energy-responses $E_{\mathrm {out},k}$ (one per anode element). Due to photon scattering and charge sharing, the response was not limited to a single anode [50]. Generally one or a few anodes produced substantially higher values of $E_{\mathrm {out},k}$ than others. As the technical specifications of the detector module indicated that only one anode registers a count per impinging photon, a post-processing step was included in which the anode with the largest value of $E_{\mathrm {out}}$ was assigned to win the photon history, while $E_{\mathrm {out},k}$ from all other anodes were discarded. The signal from the winning anode was used to record a count (scaled by the photon weight) in the energy spectrum. These initial spectra did not incorporate any energy resolution effects beyond the photopeak widening caused by the low-energy tails. Furthermore, as $\eta _k$ had values below unity ($\eta _k < 0.9$ in Fig. 2), the photopeaks tended to shift downwards. To take these factors into account, two subsequent steps were applied. In the first step, the spectrum was re-sampled according to a linear energy-calibration:

8 $$\begin{aligned} E_{\mathrm {corr}} = a_0 + a_1 \cdot E_{\mathrm {init}}, \end{aligned}$$

where $E_{\mathrm {init}}$ was the energies of the uncalibrated spectrum, and $E_{\mathrm {corr}}$ were the new energies calculated such that the photopeaks were aligned with the corresponding photon emission energies. The second step aimed at mimicking additional energy resolution effects by applying a Gaussian smoothing with an energy-dependent full width at half maximum (FWHM), as given by:

9 $$\begin{aligned} \mathrm {FWHM}(E) = b_0 + b_1 \cdot E. \end{aligned}$$

Application of Eq. 8 mimics the calibration of energy-versus-channel number used by the camera manufacturer [3]. The linear function for the FWHM (Eq. 9) was used based on the findings in Roth et al. [11].

Because the application of the energy resolution had a small effect on the peak positions, an iterative procedure was developed in which the parameters of the energy calibration (Eq. 8) were solved for given values of the energy resolution parameters (Eq. 9). Thus, the parameters $a_0,$ and $a_1$ were determined such that the photopeaks would be correctly placed after the energy resolution step (for details, see Additional file 1: Appendix C).

Energy spectra were recorded for individual anode elements in a format identical to that of the camera (“The hand-held camera” section). This format allowed spectra to be formed by adding the spectra from selected anode elements. With the anode element arrangement known, image formation was achieved by integrating all counts within a given energy-window for each individual anode-spectrum. In addition, information provided in the simulation listmode files allowed the spectra to be divided into components based on the photon classification (primary, scatter, penetration etc.) and original emission energy.

### Model tuning

“The hand-held camera” section lists specifications and parameters known for the camera and its detector module. Beyond these, other parameters such as charge mobilities, charge lifetimes, and the shapes of the electric and weighting potentials, were not known with precision. The crystal properties are known to vary throughout manufactured CZT ingots [51], and a relatively wide range of mobilities and lifetimes for electrons and holes has been reported in the literature [8, 30, 44, 52–54]. A further complication was that the features of the CIE map ($\eta _\mathrm {joint}$) and the energy resolution were not independent of each other, since the magnitude of the low-energy tails were governed by the CIE map, and in turn, these tails contributed to the energy resolution. Consequently, experimentally determined energy resolution functions [11] were not directly applicable.

**Fig. 3:**
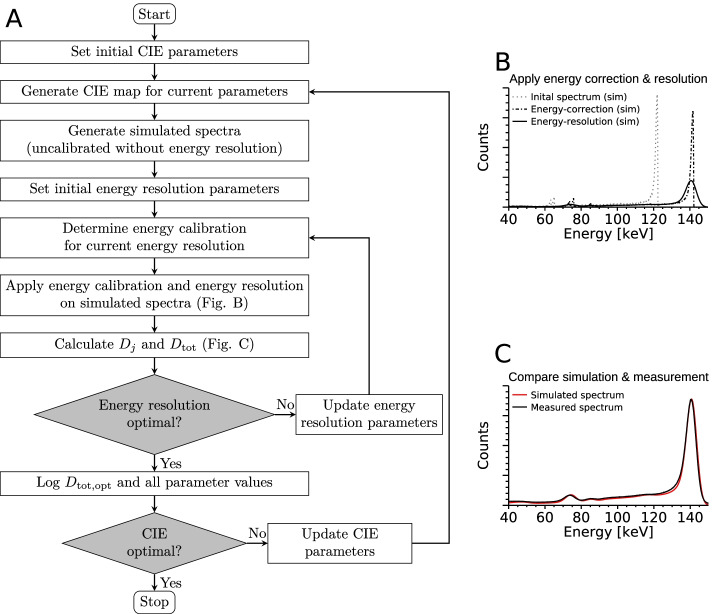
**A** Flow-chart of the model tuning procedure. **B** Example of an initial spectrum, and results after applying the energy correction and energy resolution. **C** Comparison against measured spectrum

Because of these factors, tuning of the parameter values and boundary conditions was required, with the goal of obtaining a detector model capable of replicating the experimental measurements as closely as possible. To take the large amount of experimental measurements (“Experimental measurements” section) and adjustable parameters into account simultaneously, the parameter tuning was performed as an automated procedure, illustrated in Fig. 3.

The different CIE settings listed in Table 2 were combined, and together yielded eight basic CIE configurations (Additional file 1: Appendix D). The parameter tuning procedure was executed separately for each of these configurations. In each run, the values of the configuration-associated parameters (Additional file 1: Appendix E) were optimised (outer loop in Fig. 3). Given a set of parameter values, a CIE map ($\eta _\mathrm {joint}$) was generated. For each radionuclide-collimator combination, the Monte Carlo simulated photon interactions were processed with this CIE map to form an initial spectrum (Eq. 7), the energy calibration parameters were determined, and the energy calibration and resolution applied. The energy resolution parameters were optimised separately from the CIE parameters (inner loop in Fig. 3), as the generation of a new CIE map was by far the most time-consuming step. The same energy calibration was used for all simulated spectra, and was determined using the simulation of $^{177}{\mathrm {Lu}}$ and MEGP. To enable direct comparison of measured and simulated spectra, energy calibration was also made for the measured spectra to ensure consistent photopeak positioning, which otherwise varied slightly depending on the detector temperature at measurement [11, 33]. The simulated spectra were scaled to correspond to the same activity and acquisition time as the corresponding measured spectra, and the level of agreement was determined according to

10 $$\begin{aligned} D_j = \frac{\sum _{E \in W_j}\left( f_j\cdot S_{\mathrm {sim},j}(E)-S_{\mathrm {ref,j}}(E)\right) ^2}{\left( \sum _{E \in W_j}S_{\mathrm {ref},j}(E)\right) ^2}, \end{aligned}$$

where *j* denotes one pair of simulated and measured spectra ($S_{\mathrm {sim},j}$ and $S_{\mathrm {ref},j}$). The energy interval $W_j$ was set to cover the total range of the radionuclide emissions and the operational range of the detector. The factor $f_j$ was introduced as a free parameter for simulations without a collimator (open-field) for which the system response was very sensitive to the exact source-collimator distance used at measurement. For simulations that included a collimator, $f_j$ was set to unity. Since anode elements near the edges of the detector crystal can theoretically have a different, inferior, response compared to central anodes [39], $S_{\mathrm {sim},j}$ and $S_{\mathrm {ref},j}$ were defined as the sum of the spectra from the central $14 \times 14$ anode elements.

The total level of agreement $D_{\mathrm {tot}}$ across all spectrum pairs was calculated as $D_{\mathrm {tot}} = \sum _{j} D_j$. The value of $D_{\mathrm {tot}}$ depended on the CIE map and its parameter values, as well as the energy resolution parameters (Eq. 9). The energy resolution parameters that yielded the lowest $D_{\mathrm {tot}}$ value ($D_{\mathrm {tot,opt}}$) for a given CIE map were determined using a downhill simplex method [55], with initial estimates $b_0 = 5.64\hbox { keV}$ and $b_1 = {0.00751}$ [11] (inner loop in Fig. 3). The optimal CIE map was then determined by a second simplex optimisation (outer loop in Fig. 3), in which the $D_{\mathrm {tot,opt}}$ value was considered as a function of the parameters of the CIE map. The optimal CIE map, with associated optimal energy calibration and energy resolution parameters, was thus obtained as the one yielding the best agreement (lowest $D_{\mathrm {tot,opt}}$).

## Results

The optimal CIE map was obtained using configuration A1-B2-C1 (Table 2), i.e.  using a uniform electric field, a weighting potential based on the inverse-distance-weighted transition and signal generation from electrons only. This CIE map is visualised in Fig. 2, and the associated weighting potential is shown in Additional file 1: Figure S1 (Appendix A). Results below are given for this optimal CIE map, with its optimised energy resolution parameters. A summary of the levels of agreement obtained for all configurations is provided in Additional file 1: Appendix D, and associated initial and optimal parameter values are given in Additional file 1: Appendix E.

**Fig. 4:**
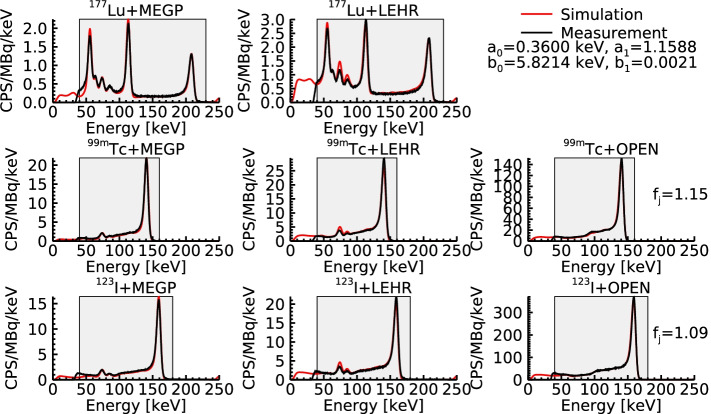
Measured and simulated energy spectra for different radionuclide-collimator combinations for the optimal CIE map. The energy resolution parameters obtained from model tuning are shown, and the shaded grey areas indicate the energy intervals considered for comparison of measured and simulated spectra (Eq. 10). The two instances where $f_j \ne 1$ are also indicated

Figure 4 shows measured and simulated energy spectra for the different radionuclide-collimator combinations used in the model tuning. Generally there is a good agreement between simulation and measurement, with small remaining deviations for x-ray peaks at lower energies for the LEHR collimator.

**Fig. 5:**
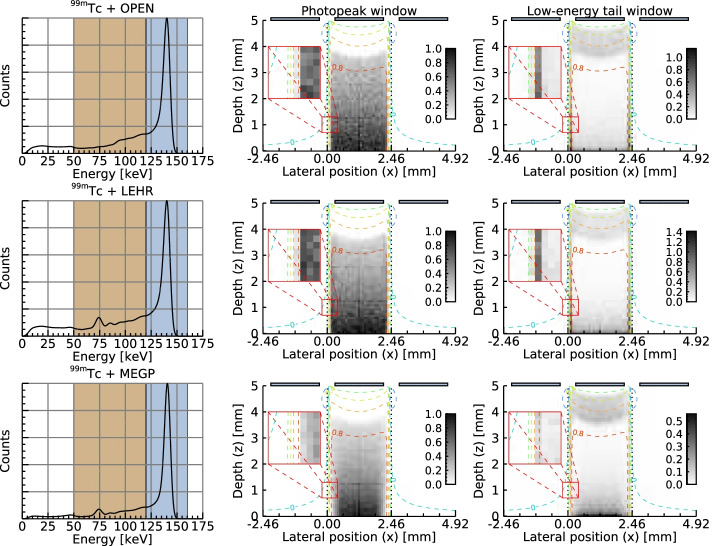
Simulated $^{99\mathrm {m}}{\mathrm {Tc}}$ spectra for the OPEN, LEHR and MEGP collimators (left column). The spectra are shown alongside interaction distributions of photons contributing to selected intervals over the photopeak and low-energy tail (middle and right columns, respectively). The interaction maps have been scaled such that the photopeak maps (middle column) have values between 0 and 1. The CIE map from Fig. 2 is overlaid on the interaction maps as isocontours. Dotted vertical lines indicate the centre of the anode gaps

Figure 5 shows simulated $^{99\mathrm {m}}{\mathrm {Tc}}$-spectra for the OPEN, LEHR and MEGP collimators. The spatial distribution of photon interaction events in the detector crystal contributing to selected spectral intervals (photopeak and low-energy tail) are illustrated besides each spectrum. All or most of the events that contribute to the photopeak occur within the relatively flat region of the CIE map ($\eta _k \simeq 0.8$ in Fig. 2), with a sharp decrease in event density where the CIE-value drops below approximately 0.7. The density of events that contribute to the photopeak decreases with increasing crystal depth due to photon attenuation. For the low-energy tail there is a contributing region near the anode, which results from the poor transport properties of holes and the small depth dependence that is still present for small-pixel devices [9]. There is also contribution from a region near the cathode, likely due to Compton and x-ray escape events as well as scattered photons and x-rays from the collimator, when present. In addition, there is contribution from regions near the lateral edges of the CIE, attributed to charge diffusion and charge sharing between neighbouring anodes [50]. The MEGP collimator shows few events in this region due to the thicker collimator septa, and as a result the low-energy tail for the MEGP collimator is lower than those of the other collimators. The remaining tail for the MEGP collimator is mainly caused by the depth-dependence of the detector.

**Fig. 6:**
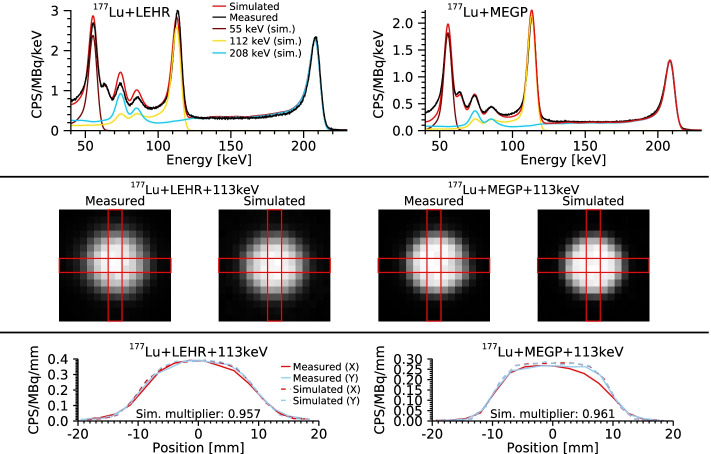
Comparison of measured and simulated data for $^{177}{\mathrm {Lu}}$ with the LEHR and MEGP collimators. Top row: energy spectra. For the simulations, a total spectrum is shown along with the individual contributions of the 55 keV, 113 keV and 208 keV photon emissions of $^{177}{\mathrm {Lu}}$. Middle row: corresponding images of the phantom for the 113 keV energy window. Red lines indicate columns and rows used to extract profiles. Bottom row: Profiles of measured and simulated images, where the simulated images have been scaled to the same total number of counts as the measured images

Further model validations are shown in Fig. 6, which includes energy spectra, images and image profiles for $^{177}{\mathrm {Lu}}$ with the LEHR and MEGP collimators. The $^{177}{\mathrm {Lu}}$ measurements are the same as in Fig. 4, and the source-collimator distances are 48 mm and 25 mm for the LEHR and MEGP collimator, respectively. The simulated spectra are separated into sub-components that reflect the contribution from the different emission energies of $^{177}{\mathrm {Lu}}$. The separation makes the interference of 208 keV photons at lower energies evident, which is more pronounced for LEHR than for MEGP. The model’s capability of producing images is also demonstrated, replicating the phantom used for measurement and using an energy window positioned over the 113 keV peak (100.5 to 120.8 keV). The profiles enable comparison of the imaging characteristics between the simulations and measurements, where a uniformity correction has been applied for measured data [11]. To aid the profile comparison, the simulated images have been scaled to the same total number of counts as for the measurements, due to small differences in system sensitivities. Slight discrepancies between measured and simulated image profiles are obtained due to difficulties of exactly replicating the position of the source in relation to the pixelated detector array and the source-collimator distance, but overall the profiles agree well.

**Fig. 7:**
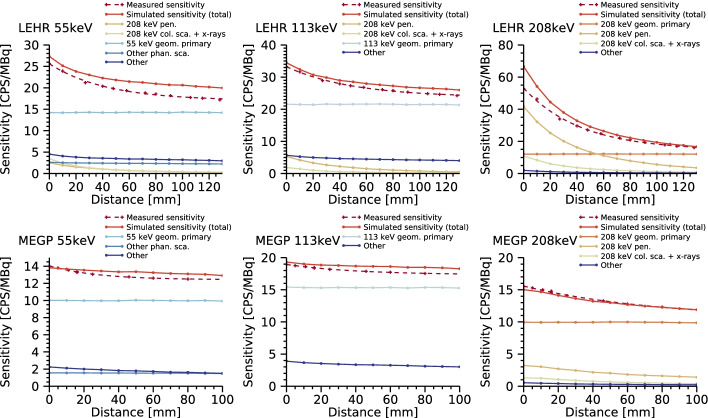
Simulated and measured system sensitivities as function of the source-collimator distance for $^{177}{\mathrm {Lu}}$ for the LEHR and MEGP collimators and energy windows positioned over 55 keV, 113 keV and 208 keV. Simulated data are connected with straight lines, for measured data a function is fitted. Simulated sensitivities are separated into sub-components based on the emission energy and events in the photon transport, including collimated primary (geom.  primary), phantom scatter (phan.  sca.), collimator penetration (pen.), collimator scatter (col.  sca.) and collimator x-rays (x-rays). Components with a low contribution are grouped together (other)

Figure 7 shows results of the system sensitivity for $^{177}{\mathrm {Lu}}$ as a function of the source-collimator distance, obtained from measurements and simulations for the LEHR and MEGP collimators. The simulated sensitivity is further separated into different sub-components, including collimated primary, phantom scatter, collimator penetration, collimator scatter and collimator x-rays, where the term primary denotes photons that pass un-scattered from the site of decay to the detection point in the crystal, and the term scatter refers to photons that have undergone Compton or Rayleigh scattering prior to detection. The system sensitivity generally decreases as a function of the source-collimator distance, irrespective of the energy window or collimator used. The simulations elucidate the cause of this behaviour: while the geometric primary component has a stationary response, the collimator penetration of primary 208 keV photons exhibits a pronounced distance dependence. This mainly affects the 208 keV window, but energy windows set at lower energies are also affected due to the low-energy tails associated with the detector. Likewise, x-rays from the collimator and 208 keV photon scatter in the collimator septa contribute to the distance-dependence. Quantitatively, the best overall agreements between simulated and measured data are obtained for the MEGP collimator and 208 keV energy window, with an average deviation obtained of 2%. The poorest overall agreement is obtained for the LEHR collimator and the 55 keV window, with an average deviation of 12%. For this energy window, errors are associated with the lower-level energy cutoff in the measured data for which exact information was not available, and also a high dependency on the modelling of the low-energy tails from both the 208 keV and the 113 keV peaks. Thus, modelling of the 55 keV peak is challenging.

## Discussion

Modelling of CZT-based gamma cameras requires more detailed considerations of the information-carrier transport and signal generation, as compared to scintillation-based detectors. To date, different models have been presented (e.g.  [20–23]), with different assumptions and approximations made. The applicability of the approximations depends on the experimental conditions for which the model is evaluated, including factors such as the collimators and radionuclides used and the energy intervals considered. The aim of this work was to develop a model for charge transport and signal generation that is applicable across different radionuclides and collimators, and to combine this model with the SIMIND Monte Carlo code for modelling of photon transport. In particular we focused on the radionuclide $^{177}{\mathrm {Lu}}$ that was found to be the most challenging among the radionuclides evaluated, owing to the three photopeaks combined with the low-energy tails characteristic of CZT detectors. The model was tuned and evaluated using the hand-held CZT camera system, for which the CZT module is identical or equivalent to those included in full-size gamma cameras (personal communication, Aharon Peretz, General Electric, retired). Figure 4 demonstrates the performance of the model, which is stable across the radionuclides and collimators tested. Overall, the obtained results agree well with measurements, allowing for simulation studies of different properties of CZT-based camera systems.

A number of assumptions and approximations have been made in order to reduce the model complexity and deal with unknown camera characteristics. As a consequence the model represents an idealised detector with a uniform response, since all anode elements are assigned identical CIE arrays and energy resolution parameters. In characterisation studies, CZT modules have been found to have a varying response between anode elements [11, 25, 33]. The CIE array and energy resolution determined as part of the model tuning thus represent an average of the detector characteristics, and differences between simulations and measurements may occur if only a few anodes are irradiated and these anodes deviate from the average. In addition, it has been reported that anode elements along the edges of the CZT module can have a different response than central anodes [39]. This effect is less relevant for the hand-held camera, as it only incorporates a single CZT module whose edge elements have limited contribution as long as the object is placed in the central FOV. Edge effects are probably more relevant for larger cameras that incorporate tiles of detector modules.

In the model tuning, the best agreement was obtained using the inverse-distance-weighted approach to define the weighting potential (Additional file 1: Appendix B). This approach is not justified by any physical processes, and should mainly be seen as a modelling tool. The reason that this approach yielded the best agreement is possibly linked to the simplifying assumptions regarding the electric potential, in particular that space charge is assumed negligible ($\rho$ in Eq. 1) and that no special consideration is made for the near-surface crystal-properties. Experiments by others indicate that such effects can influence the anode signals, but would require additional model detail [41, 42]. In our model the end results of any such effects would probably be emulated by the electric potential and weighting potential. The more physically correct approaches are those that allow for non-uniform electric fields and apply gradient-based boundary conditions for the weighting potential, i.e.  alternatives A3 and B1. However, these alternatives do not cover all electrical potentials and weighting potentials that theoretically exist, which can explain the poorer results for e.g.  configuration A3-B1-C1 (Additional file 1: Appendix D).

Signal-contribution from holes has been considered, which mainly affects the CIE-value close to the anode. By including holes, the CIE goes towards a non-zero value near the selected anode [56], while without holes it goes towards zero (Fig. 2). These two alternatives have a relatively low impact on $D_{\mathrm {tot}}$ (Additional file 1: Appendix D), and the best agreement was obtained by considering electrons only. This can be physically motivated by the low mobility and lifetime for holes, and is common in the literature [22, 30, 31, 39, 44, 50]. It should be noted that the measured spectra have a low-energy cutoff of approximately 40 keV, and it is possible that the contribution from holes would be more relevant for energies below this cutoff.

The SIMIND Monte Carlo program only handles the transport of photons. Rather than modelling the transport of secondary high-energy electrons, each photon interaction in the detector crystal has been assumed to yield a point-like charge cloud (Eq. 7). The maximum electron range is estimated to be approximately 100 ${\upmu \mathrm{m}}$, corresponding to 200 keV [57], which is in the same order as the 20 ${\upmu \mathrm{m}}$ spatial step size used in the charge transport calculations. Since the CIE map does not appreciably change over these distances (Fig. 2), the handling of the high-energy electrons is expected to have a limited impact on the results.

The model currently uses the values of $E_{\mathrm {out},k}$ (Eq. 7) to determine the winning anode for each photon history, and then applies the energy calibration and energy resolution. In practice it is reasonable to assume that the selection of a winning anode involves time-dependent anode-signals and a signal threshold, for which a more detailed approach would require a time-dependent CIE ($\eta _k\left( \mathbf {r},t\right)$) as well as timestamps for each photon interaction event. Furthermore, in practice signal noise is present at this stage, yielding randomness in the anode selection. These effects are, however, expected to have a minor influence and appear mainly for lower energies, as the induced charge must be shared near-equally between at least two anodes. A final aspect is that the currently applied energy resolution is only a function of the detector out-signal. A more detailed model would attribute the energy resolution to specific effects, including fluctuations in the number of created charges, in the number of trapped charges and electronic noise [54]. Quantification of these components would, however, likely require specialised equipment and disassembly of the camera system, and the current approximation works well for our applications.

Equations 7–9 assume that the readout-electronics have an idealised response, corresponding to a multichannel analyzer (MCA) whose channel number is linearly proportional to $E_{\mathrm {out},k}$. In effect, this assumes that the photon transport (modelled by SIMIND) and the charge transport effects (incorporated in $\eta _k$) are the major factors affecting the detectors spectral response, while effects from the preamplifier, shaping amplifier and MCA (e.g.  ballistic deficit [29]) are minor and are only included indirectly by the applied energy resolution.

Model tuning and evaluation have, as far as possible, been made based on the unaltered count levels of the measurements and the model (e.g. Eq. 10 and Figs. 4 and 7). The capability of SIMIND to replicate the system sensitivity of Anger-based cameras is already well-established (see e.g. Gustafsson et al. [58]). Some deviations between simulated and measured sensitivities are, however, expected, and include uncertainties related to the dose calibrator measurements, photon-yields and attenuation coefficients [58], as well as imperfectly replicated measurement geometries.

Results in Fig. 7 generally show good agreement between simulated and measured system sensitivities. Some remaining differences are, however, obtained, where the measured sensitivity is lower than the simulated one. This can in part be attributed to the small FOV of the camera, which increases the impact of measurement setup errors. For the simulations, the source is always perfectly aligned with the FOV centre, whereas any off-centre positioning in the measurements can lead to a loss of counts. Especially for the LEHR collimator and 208 keV there is an increasing overestimation as the source approaches the camera, which indicates an erroneously high penetration fraction. Given the good agreements obtained for the MEGP collimator for the same photon energy and the thin septa of the LEHR collimator, these results indicate that there may be an error in the technical specifications of the LEHR collimator, and adjustment of the septal thickness may be motivated. For the 113 keV and 55 keV windows the deviations are more likely attributable to the CZT model, as the contribution from septal penetration becomes smaller and the tailing-effects from higher-energy photons more pronounced.

Figure 5 shows the position-dependent response of the detector system and how this is manifested in the energy spectrum for $^{99\mathrm {m}}{\mathrm {Tc}}$. It demonstrates that the energy-tailing is caused by both the depth-dependence and the lateral edges of the CIE map. In principle, there could thus be several CIE maps with different combinations of depth-dependence and lateral shape that would have produced a given spectrum. Figures 2 and 5 demonstrate that collimators with one-to-one anode-hole matching give a consistent shielding of the gaps around all anodes, which affects the low-energy tails of the energy spectra. The matched collimators of the hand-held camera have thus provided valuable information for the model tuning, as the lateral edges of the CIE map are shielded to a varying degree. The use of several radionuclides with different emission energies (and thus different interaction-distributions depth-wise) has similarly provided information for the model. Having access to several matched collimators has likely been the most valuable factor for the model tuning, as this has resulted in drastic changes in the interaction distributions near important regions of the CIE map.

Figures 6 and 7 demonstrate how the model can be used to better understand the detector system and the energy spectrum for $^{177}{\mathrm {Lu}}$. Figure 6 concerns comparison of both energy spectra and images, while Fig. 7 shows count rates in energy windows set over the three photopeaks and as a function of source-collimator distance. The distance-dependence is of relevance for practical measurements as the depths of different objects generally vary. From the simulations it is seen that the component causing this distance-dependence is mainly collimator penetration of 208 keV photons, which, owing to the low-energy tail of the CZT detector, affects the count rate not only in the 208 keV window, but also in energy windows set at lower energies. Thus, for any quantitative measurement, this distance-dependence would need to be taken into account.

The CZT model has been valuable for understanding the general behaviour of the hand-held camera. In the future, it may help optimise the use of the camera, including the processing of images. For instance, the possibility to separate the different spectral contributions based on the original photon energy and interaction history (Fig. 7) provides a tool for development and evaluation of scatter-correction methods that can account for energy-tailing, see e.g. Kacperski et al. [10].

Our future aims include evaluation and application of the model for large-FOV CZT cameras. However, for this stage of model development, the availability of several collimators and list mode data proved to be essential for detailed model tuning and evaluation, and the compact system was found to be very useful as an experimental system.

## Conclusion

A CZT detector model has been developed and tuned to closely reflect the response of a CZT-based gamma camera. The model is based on a pre-computed numerical solution to the three-dimensional charge-transport and signal-induction equations, which is coupled to the photon-transport of the SIMIND Monte Carlo program. Model evaluation is made against measured energy spectra and images across several radionuclides and collimators, with good agreements obtained. The model provides insight to the behaviour of the camera system, particularly regarding $^{177}{\mathrm {Lu}}$ measurements, and will be useful for future optimisation of the camera application.

## Supplementary Information

**Additional file 1. Appendix A**: provides a more thorough overview of the charge transport and signal induction theories. **Appendix B** describes the inverse-distance-weighted approach to defining the weighting potential on the anode-side of the detector crystal. **Appendix C** describes the energy-calibration procedure. **Appendix D** presents the results of the model tuning for each configuration, and provides an example of the results for one configuration with poorer agreements. **Appendix E** summarises the tunable parameters available under each configuration.

## Data Availability

The datasets used and analysed during the current study are available from the corresponding author on reasonable request. The developed CZT model will be incorporated in a future release of SIMIND (homepage: https://simind.blogg.lu.se/).

## Abbreviations

CZT: Cadmium zinc telluride
FOV: Field of view
LEHR: Low energy high resolution
MEGP: Medium energy general purpose
FWHM: Full width at half maximum
MCA: Multichannel analyzer
CIE: Charge induction efficiency
SPECT: Single-photon emission computed tomography
