# Supplementary material for: Monte Carlo modelling of a compact CZT-based gamma camera with application to 177Lu imaging
Source: EJNMMI Phys. 2022 May 8;9:35. doi: 10.1186/s40658-022-00463-1 (PMC9081070; doi:10.1186/s40658-022-00463-1)
Supplement: Supplementary file 1 — Additional file 1. Appendix A: provides a more thorough overview of the charge transport and signal induction theories. Appendix B describes the inverse-distance-weighted approach to defining the weighting potential on the anode-side of the detector crystal. Appendix C describes the energy-calibration procedure. Appendix D presents the results of the model tuning for each configuration, and provides an example of the results for one configuration with poorer agreements. Appendix E summarises the tunable parameters available under each configuration. [file 40658_2022_463_MOESM1_ESM.pdf]

Appendix A: Theory on signal induction and charge transport

A.1 Charge induction

The Shockley-Ramo theorem can be used to calculate the readout-signal from semiconductor detectors [26–28]. The theorem can be applied to any detector type that uses an electric field over a volume to detect charged particles created from ionising radiation, and states that a point charge q moving at a velocity $\vec{v}$ induces a current i_k on an electrode (numbered k) according to

$$i_k = q \cdot \vec{v} \cdot \vec{E}_k, \tag{S1}$$

where $\vec{E}_k$ is the so-called weighting field of electrode k. The weighting field does not affect matter in the same manner as e.g. an electric field, and instead constitutes a tool for signal calculation. The charge velocity $\vec{v}$ is related to the electric field $\vec{E}$ in the detector according to

$$\vec{v} = \pm\mu \cdot \vec{E}, \tag{S2}$$

where μ is the charge mobility, and the sign depends on the sign of the particle charge. The fields $\vec{E}$ and $\vec{E}_k$ are given as gradients of an electric potential φ (unit V) and a weighting potential φ_k (unitless), respectively.

$$\vec{E} = \quad -\nabla\varphi \tag{S3}$$

$$\vec{E}_k = \quad -\nabla\varphi_k. \tag{S4}$$

The electric potential φ is given by the solution to Gauss's law

$$\nabla^2\varphi = \frac{\rho}{\varepsilon}, \tag{1 \textit{in main}}$$

where ρ is the charge density in the detector volume and ε is the permittivity of the detector material [29, 30]. The weighting potential φ_k is calculated similarly, but with the space charge set to zero, using the equation

$$\nabla^2\varphi_k = 0. \tag{2 \textit{in main}}$$

An example of a weighting potential for a single anode in a pixelated-anode-type detector is illustrated in Figure S1.

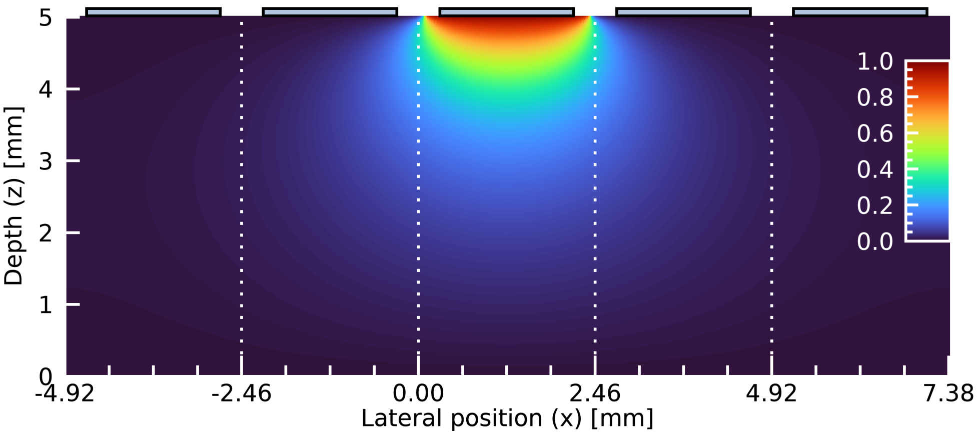

Figure S1: Weighting potential cross-section corresponding to one anode for a 5 mm thick pixelised CZT detector, and a lateral distance corresponding to five anodes indicated at the top. Dotted lines indicate the centre of the gaps between anodes. The weighting potential has been calculated following section 2.4, using the specifications in section 2.2.

Integration of the current $i_k(t)$ during the period that the charge exists in the detector volume gives the induced charge $\Delta Q_k(t)$ on the electrode

$$\Delta Q_k(t) = \int_0^t i_k(t')\,\mathrm{d}t'. \tag{S5}$$

The induced charge can be considered as a function of the integration time t, as well as the point $\vec{r}_0$ where the charge was initially created, i.e. $\Delta Q_k(t) = \Delta Q_k(\vec{r}_0, t)$. The charge induction efficiency (CIE) η is defined as the induced charge divided by the magnitude of the initially created charge q according to $\eta_k\left(\vec{r}_0, t\right) = Q_k(\vec{r}_0, t)/|q|$.

A.2 Charge transport

Equation S1 applies to a point charge. Groups of charge carriers will tend to disperse into charge clouds due to diffusion. The distribution of charge carriers will be denoted as $n\left(\vec{r}, t\right)$ (electrons) and $p\left(\vec{r}, t\right)$ (holes). While the motion of a point charge can be described by equation S2, the motion of

charge clouds are described by the following differential equations [19, 30, 31]:

$$\frac{\partial n}{\partial t} = -\nabla \cdot (\mu_n n \nabla \varphi) + \nabla \cdot (D_n \nabla n) + G_n - R_n, \tag{S6}$$

$$\frac{\partial p}{\partial t} = \nabla \cdot (\mu_p p \nabla \varphi) + \nabla \cdot (D_p \nabla p) + G_p - R_p. \tag{S7}$$

The term $\nabla \cdot (\mu_x x \nabla \varphi)$ (where x denotes either n or p) is analogous to equation S2 and describes the motion of the charge cloud due to the electric field. The term $\nabla \cdot (D_x \nabla x)$ describes motion due to diffusion, where D_x is the diffusion constant. The terms G_x and R_x describe the creation of new charges (e.g. by ionizing radiation interactions) and recombination of existing charges, respectively. Charge recombination can be described as $R_x = x/\tau_x$, where τ_x is the charge carrier lifetime. For ionizing radiation interactions, the electrode signal caused by a single point-like charge generated at an arbitrary point $\vec{r}_0$ within the detector volume may be of interest. The response to such an event, at $t = 0$, can be modelled using $G_x = \delta\left(|\vec{r} - \vec{r}_0|\right) \cdot \delta\left(t\right)$, where $\delta(\cdot)$ is the Dirac delta function.

The diffusion constant D_x is given by

$$D_x = \frac{k_B \cdot T}{q} \cdot \mu_x, \tag{S8}$$

where k_B is the Boltzmann constant, T is the absolute temperature of the detector and q is the particle charge [32].

For charge clouds, equation S1 and S5 can be expressed as

$$\Delta Q_{x,k}(t) = q \cdot \int_0^t \int_{\vec{r} \in \Omega} x\left(\vec{r}, t'\right) \cdot \mu_x \cdot \nabla \varphi\left(\vec{r}\right) \cdot \nabla \varphi_k\left(\vec{r}\right) \mathrm{d}\Omega \, \mathrm{d}t', \tag{4 \textit{in main}}$$

where Ω is the detector volume [19, 22, 30, 31]. Calculation of $x(\vec{r}, t)$ and $\eta_{x,k}\left(\vec{r}_0, t\right)$ for a large number of starting positions $\vec{r}_0$ directly using equations S6, S7 and 4 is computationally expensive. A more efficient method for computing η is to use an adjoint method [19, 31], wherein the differential equations

$$\frac{\partial n^+}{\partial t} = \mu_n \nabla \varphi \cdot \nabla n^+ + \nabla \cdot (D_n \nabla n^+) + G_n^+ - n^+/\tau_n, \tag{S9}$$

$$\frac{\partial p^+}{\partial t} = -\mu_p \nabla \varphi \cdot \nabla p^+ + \nabla \cdot (D_p \nabla p^+) + G_p^+ - p^+/\tau_p, \tag{S10}$$

are solved instead of equation S6 and S7. For generation terms defined as $G_n^+ = \mu_n \nabla\varphi \cdot \nabla\varphi_k$ and $G_p^+ = \mu_p \nabla\varphi \cdot \nabla\varphi_k$, it can be shown that $n^+(\vec{r}, t) = \eta_{n,k}(\vec{r}, t)$ and $p^+(\vec{r}, t) = \eta_{p,k}(\vec{r}, t)$ [19, 31]. This means that $\eta_{n,k}(\vec{r}, t)$ and $\eta_{p,k}(\vec{r}, t)$ can be determined for all starting positions simultaneously by solving equation S9 and S10 once, rather than repeatedly solving equation S6 and S7 and evaluating equation 4 for all possible $\vec{r}_0$.

Appendix B: Parameterised weighting potential

This section describes the process used to define the weighting potential (φ_k) across the anode-side of the detector crystal in the inverse-distance-weighted transition method.

The boundary condition given to the weighting potential at the crystal surface covered by anodes is $\varphi_k = \varphi_{\text{Anode}}$. The function $\varphi_{\text{Anode}}(x, y)$ is defined on a Cartesian coordinate system (x, y) aligned with the anode array arrangement (Figure S2). The origin is located in the middle of the inter-anode gap near the lower left corner of the selected anode (electrode k). The pixel pitch q, contact pad width w and gap length g are assumed to be identical in the x and y directions.

The distance $f(d)$ to anode A in each dimension is calculated as

$$f(d) = \begin{cases} 0 & : \ \frac{g}{2} \le d \le q - \frac{g}{2} \\ \min\left(\left|d - \frac{g}{2}\right|, \left|d - \left(q - \frac{g}{2}\right)\right|\right) & : \ \text{otherwise,} \end{cases} \tag{S11}$$

where d is a x- or y-coordinate. The relative distances to the selected anode in each dimension are defined as

$$x_{\text{d}} = \frac{f(x)}{g \cdot s}, \tag{S12}$$

$$y_{\text{d}} = \frac{f(y)}{g \cdot s}. \tag{S13}$$

Here, s is a parameter introduced in order to make the ‘width’ of the weighting potential and CIE map adjustable. Normally, $s = 1$, but values between 0 and 1 can be used to obtain a ‘narrower’ CIE map. For all positions in contact with the selected anode, $x_d = y_d = 0$. Positions where $0 < x_{\text{d}}, y_{\text{d}} < 1$ are interpreted as lying in the gap between the selected anode and its neighbours. Positions where $x_{\text{d}} \ge 1$ or $y_{\text{d}} \ge 1$ are interpreted as lying on the other anodes, or in the gaps between them.

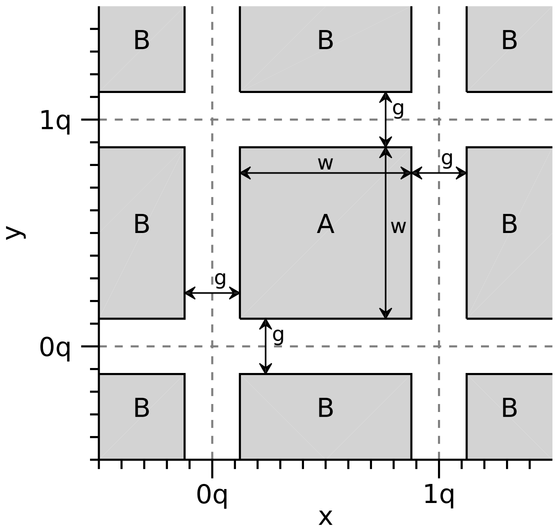

Figure S2: Illustration of coordinate system and anode layout. Gray squares represent anodes. Dashed lines indicate the middle of the inter-anode gaps. The selected anode is labelled A, while other anodes are labelled B.

By definition, φ_{Anode} should have the value 1 on the selected anode, and the value 0 on all other anodes. φ_{Anode} is further assumed to have the value 0 in all inter-anode gaps that are not directly adjacent to the selected anode ($x_{\mathrm{d}}, y_{\mathrm{d}} > 1$). In the adjacent gap, a transition from 1 and 0 is achieved using inverse-distance weighting.

Interpolation between anode edges defined by line segments is handled in each dimension separately. For positions within the gap between the sides of two anodes ($x_{\mathrm{d}} = 0$ and $0 < y_{\mathrm{d}} < 1$, or $0 < x_{\mathrm{d}} < 1$ and $y_{\mathrm{d}} = 0$), only one interpolation is necessary. For positions within a gap between the corners of four anodes ($0 < x_{\mathrm{d}}, y_{\mathrm{d}} < 1$), two interpolations (analogous to bi-linear interpolation) are applied.

A distance-dependent function W is defined as

$$W(l) = \frac{1}{l^p}, \tag{S14}$$

where l is a distance along one dimension and p is a parameter that controls

the steepness of the transition. The weighting function I is defined as

$$I(W_0, P_0, W_1, P_1) = \frac{W_0 \cdot P_0 + W_1 \cdot P_1}{W_0 + W_1}. \tag{S15}$$

Using W and I, $\varphi_{\text{Anode}}(x, y)$ is defined as

$$\varphi_{\text{Anode}}(x, y) = \begin{cases} 1 & : \quad x_{\text{d}} = 0 \quad \wedge \quad y_{\text{d}} = 0 \\ 0 & : \quad x_{\text{d}} \geq 1 \quad \vee \quad y_{\text{d}} \geq 1 \\ I(W(y_{\text{d}}), 1, W(1 - y_{\text{d}}), 0) & : \quad x_{\text{d}} = 0 \quad \wedge \quad 0 < y_{\text{d}} < 1 \\ I(W(x_{\text{d}}), 1, W(1 - x_{\text{d}}), 0) & : \quad 0 < x_{\text{d}} < 1 \quad \wedge \quad y_{\text{d}} = 0 \\ I(W(x_{\text{d}}), I(W(y_{\text{d}}), 1, W(1 - y_{\text{d}}), 0), W(1 - x_{\text{d}}), 0) & : \quad 0 < x_{\text{d}} < 1 \quad \wedge \quad 0 < y_{\text{d}} < 1. \end{cases} \tag{S16}$$

The result and the possibility for adjusting the transition steepness are illustrated in Figure S3.

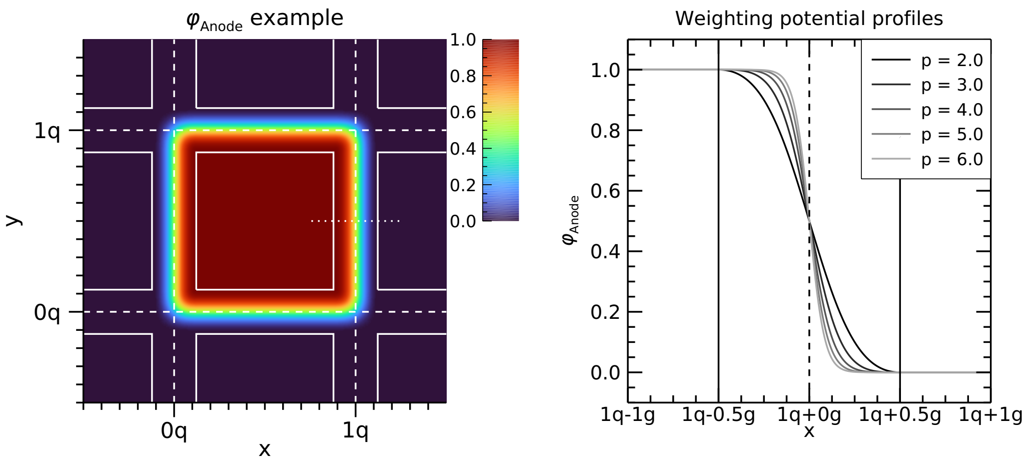

Figure S3: Left: Illustration of $\varphi_{\text{Anode}}(x, y)$ for $p = 2$. Right: Weighting potential profiles for different values of p. Vertical lines indicates the beginning, middle and end of the inter-anode gap. The dotted line in the left figure indicates the location of the drawn profiles.

B.1 Remarks

The starting point for the inverse-distance-weighted transition is the approach by Mayer et al. [52]. In their work, the weighting potential was as-

sumed to decrease linearly from unity at the edges of one electrode to zero at the edges of the neighbouring electrodes. This assumption of a linear transition was later described as 'quite arbitrary' [43], but nevertheless seeded the idea that a non-linear user-defined parametrised transition could be useful for model-development.

Additional comparisons can be made against the charge sharing method of Pretorius et al. [23] and its current SIMIND-implementation (version 6.1 and later). For instances where electrons reach the gap between neighbouring anodes, this method distributes the induced charge between the anodes using a bi-linear interpolation, somewhat analogous to a bi-linear weighting potential transition (linear between edges, bi-linear near corners). The inverse-distance-weighted method, compared to a linear function, yields a steeper transition within a narrow region. In effect, from the charge-sharing perspective of Pretorius et al. [23], the inverse-distance-weighted transition would have a preference towards assigning most of the induced charge to a single anode.

It should be noted that the proper method for obtaining the weighting potential is to consider it both inside and outside the crystal volume, and impose a boundary condition of a continuous gradient (multiplied by the local permittivity) across the gaps [39, 43]. Factors such as thin surface-layers of imperfect crystal material and a crystal substrate could additionally be considered within this framework [41, 42]. This introduces several parameters which system end-users generally have insufficient data on. Configuration B1 (zero-gradient) is a variant of this boundary condition, and is derived as an approximation with the condition that the crystal is surrounded by air [39]. Within this context, the inverse-distance-weighted transition can be regarded as a simplified method that can produce reasonable transitions.

Appendix C: Energy calibration procedure

The iterative procedure implemented to determine the energy calibration parameters (equation 8) is illustrated in Figure S4.

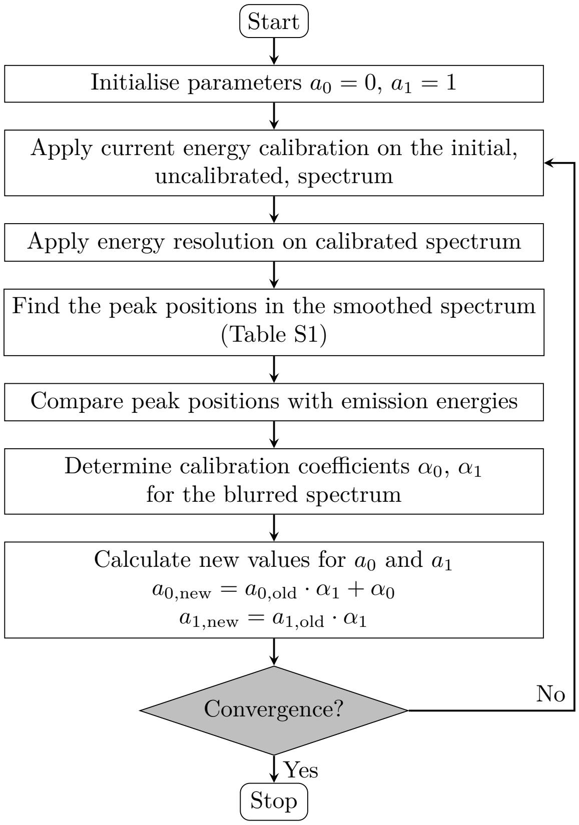

Figure S4: Flowchart illustrating the processes involved to determine the energy calibration.

Table S1: Photopeaks used for energy correction and intervals used to find the peak positions.

Nuclide	Energy [keV]	Search interval [keV]
^{177}Lu	55.8	45–60
^{177}Lu	112.9	95–120
^{177}Lu	208.4	180–220
^{99m}Tc	140.5	110–150
^{123}I	159.0	130–170
Lead x-ray[1]	73.6	50–80

[1] Yield-weighted average of $K_{\alpha 1}$ and $K_{\alpha 2}$. Considered for ^{99m}Tc and ^{123}I spectra where the MEGP or LEHR collimator were used.

Appendix D: CIE configuration results

Table S2 shows the optimal agreements for each model configuration. Figure S5 illustrates the CIE maps associated with these agreement values. Figure S6, demonstrates the measured and simulated energy spectra underlying one of the poorer agreements (configuration A3-B1-C1), and constitutes a counterpart to Figure 4. The simulated spectra correspond to the optimal CIE map (shown in Figure S5) with its optimal energy resolution parameters. Compared to configuration A1-B2-C1 (Figure 4), a greater proportion of counts is located in the tails. This effect is less pronounced in the spectra obtained with the MEGP collimator, indicating a link to the lateral CIE edges.

Table S2: Optimal agreements (lowest $D_{\mathrm{tot,opt}}$) obtained for the different configurations given in Table 2. Agreements are expressed relative to the globally best result.

Configuration	Agreement value
A1-B1-C1	6.692
A1-B1-C2	5.376
A1-B2-C1	1.000
A1-B2-C2	1.019
A3-B1-C1	2.409
A3-B1-C2	2.038
A3-B2-C1	1.021
A3-B2-C2	1.009

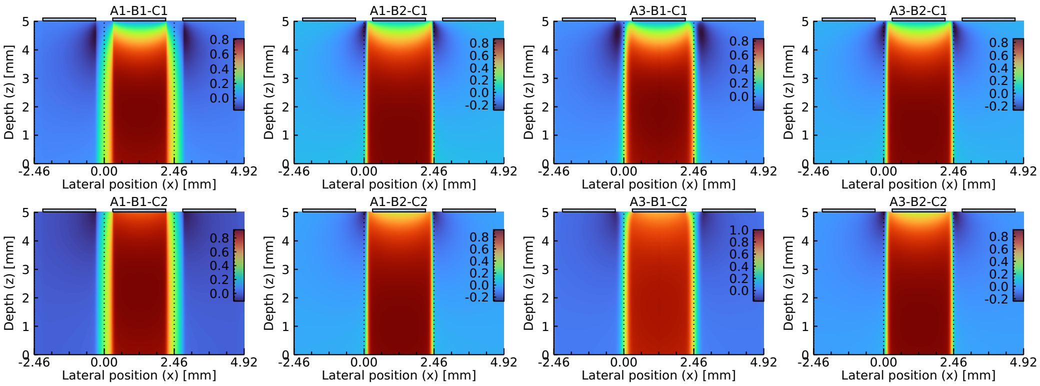

Figure S5: Cross-sections of the optimal CIE maps under each configuration.

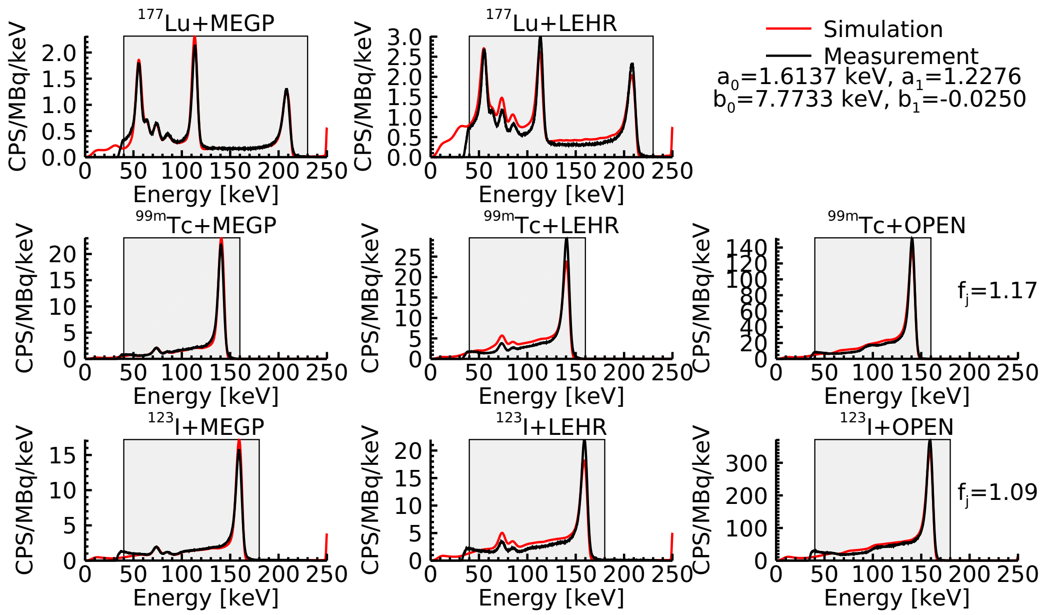

Figure S6: Measured and simulated energy spectra for different radionuclide-collimator combinations for the optimal CIE map under configuration A3-B1-C1. The energy resolution parameters obtained from model tuning are also shown, and the shaded grey areas indicate the energy intervals considered for comparison of measured and simulated spectra (equation 10).

Appendix E: Tunable parameters

Table S3 presents the parameters tuned for the different configurations of the CIE calculation procedure. Table S4 presents the initial parameter values used and the final optimal values for each configuration.

Table S3: Tunable parameters associated with the different CIE calculation configurations.

Alternative	Affected component	Tunable parameters			#
A3	Electric potential (weighted average of the results from A1 and A2)	m			1
B2	Weighting potential (inverse-distance weighted transition)	p	s		2
C1	Electron transport	$\mu_e \cdot \tau_e$	c_e		2
C2	Electron and hole transport	$\mu_e \cdot \tau_e$	c_e	$\mu_h \cdot \tau_h$	3

Parameter description:

- For alternative A3 two electric potentials, φ_{A1} and φ_{A2}, are calculated using the configurations of alternative A1 and A2, respectively. The final electric potential used in subsequent calculations is then calculated as $\varphi_{\mathrm{A3}} = m \cdot \varphi_{\mathrm{A1}} + (1-m) \cdot \varphi_{\mathrm{A2}}$.

- The parameter c_e is a scale factor for the diffusion constant (D_e). This parameter is motivated by uncertainties in detector temperature, and affects the lateral edge sharpness of the CIE. A corresponding multiplier is not used for holes due to their short ranges.

Table S4: Initial (I) and final (F) parameter values for the optimisation of each configuration.

Configuration	$\mu_e \cdot \tau_e$ $10^{-6}\mathrm{cm}^2/\mathrm{V}$ I → F	$\mu_h \cdot \tau_h$ $10^{-6}\mathrm{cm}^2/\mathrm{V}$ I → F	c_e Unitless I → F	s Unitless I → F	p Unitless I → F	m Unitless I → F
A1-B1-C1	3840 1391		1.00 0.43			
A1-B1-C2	3840 2963	50 203	1.00 1.02			
A1-B2-C1	3840 2476		1.00 1.03	0.85 0.75	6.00 9.56	
A1-B2-C2	3840 3882	50 57	1.00 1.18	0.85 0.70	6.00 6.11	
A3-B1-C1	3840 1480		1.00 1.17			0.50 0.41
A3-B1-C2	3840 3434	50 131	1.00 0.92			0.50 0.53
A3-B2-C1	3840 2171		1.00 0.98	1.00 0.71	1.50 4.79	0.50 0.99
A3-B2-C2	3840 3332	50 51	1.00 0.84	1.00 0.70	1.50 3.79	0.50 1.00
